# Cohabitation of *Piscirickettsia salmonis* genogroups (LF-89 and EM-90): synergistic effect on growth dynamics

**DOI:** 10.3389/fcimb.2023.1253577

**Published:** 2023-10-25

**Authors:** Gabriela Carril, Hanne C. Winther-Larsen, Marie Løvoll, Henning Sørum

**Affiliations:** ^1^ Department of Paraclinical Sciences, Faculty of Veterinary Medicine, Norwegian University of Life Sciences, Ås, Norway; ^2^ Department of Pharmacology and Pharmaceutical Biosciences, Department of Pharmacy, University of Oslo, Oslo, Norway; ^3^ VESO Vikan, Namsos, Norway

**Keywords:** intracellular pathogen, Piscirickettsiosis, SRS, co-infection, virulence factor, biofilm, Atlantic salmon

## Abstract

*Piscirickettsia salmonis*, the biological agent of Salmonid Rickettsial Septicemia (SRS), is a facultative intracellular bacterium that can be divided into two genogroups (LF-89 and EM-90) with different virulence levels and patterns. Studies have found co-infection of these genogroups in salmonid farms in Chile, but it is essential to assess whether this interaction within the host is related to virulence and changes in pathogen dynamics. In this study, we studied four isolates from EM-90 and one LF-89 isolate chosen based on their genomic differences. The aim was to evaluate how co-cultivation affects bacterial growth performance and virulence factor expression in Atlantic salmon (*Salmo salar*) *in vitro* and *in vivo*. *In vitro* results using FN2 medium, showed a similar growth curve between co-cultures of LF-89 and EM-90 compared to EM-90 monocultures. This was explained by the higher ratio of EM-90 to LF-89 in all co-cultures. When evaluating the expression of virulence factors, it was discovered that the *luxR* gene was expressed only in EM-90-like isolates and that there were significant differences between mono- and co-cultures for *flaA* and *cheA*, suggesting a response to cohabitation. Moreover, during *in vivo* co-cultures, transcriptomic analysis revealed an upregulation of transposases, flagellum-related genes (*fliI* and *flgK*), transporters, and permeases that could unveil novel virulence effectors used in the early infection process of *P. salmonis*. Thus, our work has shown that cohabitation of *P. salmonis* genogroups can modulate their behavior and virulence effector expression. These data can contribute to new strategies and approaches to improve the current health treatments against this salmonid pathogen.

## 1 Introduction

*Piscirickettsia salmonis* is a facultative intracellular Gamma-proteobacteria that in Chile causes the systemic disease Salmonid Rickettsial Septicemia (SRS) in several salmonid species such as Atlantic salmon (*Salmo salar*), rainbow trout (*Oncorhynchus mykiss*), and salmon coho (*Oncorhynchus kisutch*) (SERNAPESCA, 2022a). In other important salmon-farming countries such as Norway (Olsen et al., 1997), Canada (Brocklebank et al., 1992), Scotland (Grant et al., 1996), and Ireland (Rodger and Drinan, 1993), *P. salmonis* is considered a potential emerging pathogen (Long and Jones, 2021).

In Chile, SRS was the cause of mortality in 54.2% of Atlantic salmon that died during the first half of 2022 (SERNAPESCA, 2022a), resulting in annual economic losses of US$700 million for the aquaculture industry (Maisey et al., 2017). To deal with the SRS, the available treatments consist mainly in the use of antibiotic (e.g., florfenicol) reaching up to 238.2 tons in the same period (SERNAPESCA, 2022b). In addition, 28 commercial vaccines have been approved for use against SRS (SAG, 2022), but missing the expected efficacy by the industry (Figueroa et al., 2022). This could be due to how this pathogen evades the salmon immune response owing to its intracellular nature. When replicating within the cytoplasmic vacuoles of macrophages (Rozas-Serri, 2022), it inhibits phagosome–lysosome fusion through the secretion of virulence effectors using the Dot/Icm secretion system (Gomez et al., 2013) and induces cell apoptosis (Rojas et al., 2010).

*P. salmonis* is the only species belonging to the genus *Piscirickettsia* (Fryer et al., 1992) and is closely related to the genera *Coxiella*, *Francisella*, and *Legionella* genera (Wilhelm et al., 2006). Based on several genomic and phylogenetic studies*, P. salmonis* can be divided into two genogroups or subspecies (LF-89 and EM-90) (Mauel et al., 1999; Casanova et al., 2003; Reid et al., 2004; Bohle et al., 2014; Mandakovic et al., 2016; Otterlei et al., 2016; Nourdin-Galindo et al., 2017; Isla et al., 2019). It has been demonstrated that these two genogroups have different virulence levels and patterns (Rozas-Serri et al., 2017; Saavedra et al., 2017). In addition, recently the co-infection by both genogroups has been proven in Atlantic salmon at farm, fish, and tissue levels (Rozas-Serri et al., 2023).

The high salmon mortality rate caused by SRS in Chile could be related to changes in the disease dynamics of this multiple-genotype infection through competitive interactions (Kotob et al., 2016) and to the selection of strains with higher virulence, as a result of intensive farming conditions (Sundberg et al., 2016). Interestingly, modulation of the dynamics of infections has already been reported in other salmonid pathogens such as *Moritella viscosa* (etiological agent of winter-ulcer disease), whose transcription profile may be altered during co-cultures with *Aliivibrio wodanis* (Hjerde et al., 2015). Therefore, evaluating the complex interactions between *P. salmonis* LF-89 and EM-90 is important, because it could change the approach and strategies to control SRS. 

Thus, the aim of this study was to investigate how the cohabitation of both genogroups, LF-89 and EM-90 can modulate their growth performance and virulence factors under *in vitro* and *in vivo* conditions in Atlantic salmon. This contributes to a better understanding of the SRS infection process and development of more precise treatments.

## 2 Materials and methods

### 2.1 *P. salmonis* isolates

Five *P. salmonis* isolates from Atlantic salmon in Chile provided by Prof. Sergio Marshall (Laboratory of Genetics and Molecular Immunology, Pontifical Catholic University of Valparaíso, Chile) were used in this study: one from the LF-89 genogroup (Psal-013) and four from the EM-90 genogroup (Psal-051, Psal-068, Psal-163, and Psal-182) (**Table 1**). The bacteria were stored at −80°C with glycerol and 20% fetal bovine serum (FBS) stocks and routinely cultured on cysteine heart agar plates (CHAB) supplemented with bovine blood (Norwegian Veterinary Institute, Oslo, Norway) and in FN2 broth medium (Bravo and Martinez, 2016) with shaking at 100 rpm and incubated at 18°C.

**Table 1:** *Piscirickettsia salmonis* isolates used in this study.

Strain ID	Host	Organ	Region/Zone	Year	Genogroup
**Psal-013**	*Salmo salar*	Kidney	Región de Los Lagos	2012	LF-89
**Psal-051**	*Salmo salar*	Liver	Región de Aysén	2017	EM-90
**Psal-068**	*Salmo salar*	Kidney/Liver/Brain	Región de Aysén	2011	EM-90
**Psal-163**	*Salmo salar*	Kidney	Región de Los Lagos	2011	EM-90
**Psal-182**	*Salmo salar*	Liver	Región de Aysén	2018	EM-90

### 2.2 *In vitro* co-culture of *P. salmonis* isolates

To measure growth, the FN2 broth medium was used. Freshly grown bacterial cultures were adjusted to an initial OD of 0.02 at 600 nm and transferred into a 250 mL sterile flask with broth medium (25 mL). The flasks were maintained in agitation (100 rpm) at 18°C for 6 days for mono- and co-culture growth. The start point of the genogroup proportion for the co-cultures was a 1:1 ratio.

Each culture assay was performed in four independent replicates. Growth data were measured using a PV4 Visible Spectrophotometer (VWR) every 24 h. In addition, 1 mL samples were taken at the same time to extract total DNA, and at 48 h, 96 h, and 120 h a 500 μL sample was taken for further RNA extraction to monitor changes in *P. salmonis* gene expression during mono- and co-culture growth.

To check for contamination and viability, 100 μL of each liquid culture were replated on CHAB agar plates and incubated at 18°C for 10 days to evaluate colonies and further DNA extraction to perform an RFLP-PCR analysis specific for *P. salmonis* (Aravena et al., 2020).

### 2.3 DNA extraction and growth rate of *P. salmonis* genogroups

All the samples taken from bacterial growth kinetics were first centrifuged at 10,000×*g* for 10 min. The pellets were then resuspended in lysis buffer (1 M Tris–HCl, 0.2 M EDTA, 10% Triton X-100) with lysozyme and incubated for 30 min at 37°C. Protein kinase (Qiagen, Hilden, Germany) was added and incubated at 56°C for 30 min. DNA was extracted using QIAcube and QIAGEN DNeasy® Blood & Tissue Kit (Qiagen) according to the manufacturer’s instructions and stored at −20°C until use. A NanoDrop™ ND‐1000 spectrophotometer (Thermo Scientific) was used to determine the concentration and quality of DNA.

To determine the growth rate of *P. salmonis* genogroups, DNA samples from the co-cultures were used in triplicate for qPCR assays using an AriaMx Real-Time PCR System (Agilent Technologies). Specific primers targeting each genogroup’s unique gene were designed (**Table 2**). The PCR reaction mixture contained 10 μL of Maxima SYBR Green Master Mix (Thermo Scientific), 300 nM of each specific forward and reverse primer and 15 ng of DNA template in a total volume of 20 μL. The thermal profile started with an initial denaturation at 95°C for 3 min followed by 40 cycles at 95°C for 15 s and 60°C for 60 s, and a final melting curve between 60°C and 95°C. For all primers, efficiencies were established using the equation E = 10^(−1/slope)^. Relative quantification was estimated using threshold cycle (ct) values, normalizing the Ct of the target gene to the housekeeping gene for the same sample (at the same time), and then calculating the ratio between genogroups in fold changes of EM-90 compared to LF-89.

**Table 2:** Primers used in this study.

Gene	Primers (5’-3’)	Description	Tm
*glyA*	F: CGCGTACCATTGCAGATTTCGACC R: GCTTCTAGCACACGCGGACTCG	Housekeeping gene	60°C
*LF013*	F: AAAGAGCCCTGACCAAACAA R: CCCTGAGTTGTCAACAGCAA	LF-89-like	60°C
*EM182*	F: CTCTACGCATGGGAACAGTG R: CACCACCAACAACACTACCG	EM-90-like	60°C
*flaA*	F: GCATGGCACTTTCAGTTGTAACCAAC R: CAAGGCCAATACTCGCATCCGCTG	Flagellin	60°C
*relA*	F: CATATCTTGACCACGACGGATAG R: GGTGCAGTAAGCTGTGAAGTA	(p)ppGpp inducer	60°C
*cheA*	F: CACCACTTCTTCTTGACCTACA R: GGTTGATTAAAGACTCACCGAAAG	Chemotaxis activity	60°C
*luxR*	F: CGCTCGGACAATCACCTAAA R: CCTAAGGCATAGCCTAGTTCAAT	N-acylhomoserine lactone (AHL)	60°C

### 2.4 Gene expression of target biomarkers

To evaluate the gene expression of target biomarkers in *P. salmonis* at 48 h, 96 h, and 120 h, 500 µL of each sample was centrifuged at 10,000×*g* for 10 min at 4°C to recover the pellet, which was used for RNA extraction using the Qiagen RNeasy® Mini Kit (Qiagen) according to the manufacturer’s protocol. A NanoDrop™ ND‐1000 spectrophotometer (Thermo Scientific) was used to determine the quality and quantify each RNA sample. Then cDNA was synthesized using the QuantiTect® Reverse Transcription Kit (Qiagen) following the manufacturer’s instructions.

cDNA samples were used in three technical replicates for each of the four biological replicates. Briefly, for RT-qPCR MIX, 15 ng of cDNA was used for each sample along with 10 μL of Maxima SYBR Green Master Mix (Thermo Scientific) and 300 nM of forward and reverse primers in a total volume of 20 μL. The thermal profile started with an initial denaturation at 95°C for 3 min, followed by 40 cycles at 95°C for 15 s and 60°C for 60 s, and a final melting curve between 60°C and 95°C.

Specific primers were used to establish key gene expression changes in the co-cultures compared to the monoculture using *glyA* as the normalizer (**Table 2**). For all primers, efficiencies were established using the equation E = 10^(−1/slope)^. Relative gene expression was estimated using threshold cycle (Ct) values and the Livak 2^−ΔΔCt^ method (Livak and Schmittgen, 2001).

### 2.5 Biofilm assay

Frozen stocks at −80°C from the five isolates were streaked onto cysteine heart agar plates (CHAB) supplemented with bovine blood (Norwegian Veterinary Institute, Oslo, Norway) and incubated at 18°C until growth. Single colonies of the isolates were used to inoculate 3 mL of FN2 broth, which was then incubated at 18°C. After the cultures reached the exponential phase, the optical density (OD) of each culture at 600 nm was adjusted to 0.02 to be used as an inoculum for the 96 well microplates to perform a biofilm formation assay.

Three independent experiments were performed by inoculating eight wells per isolate and co-culturing with 150 µL of the bacterial suspension in FN2 broth. FN2 medium without bacteria was used as the negative control. The microplates were incubated at 18°C with shaking at 100 rpm. After 120 h, all the medium and planktonic bacteria were carefully removed from the inoculated and control wells. Bacteria that adhered to the wells of the microplates were stained with 180 µl of crystal violet solution (0.1%) at room temperature for 10 min. The crystal violet (CV) solution was removed, and the walls were rinsed thoroughly with sterile distilled water. The microplates were turned upside down and dried for 1 h. For CV solubilization, absolute ethanol (200 µL/well) was added and left at room temperature for 10 min. The resulting solutions were homogenized by repeated pipetting before reading the microplates at 620 nm using a PV4 Visible Spectrophotometer (VWR).

### 2.6 *In vivo* co-culture of *P. salmonis* genogroups for RNA isolation and transcriptomics sequencing

One isolate from each genogroup was selected for *in vivo* cultivation, Psal-013 (LF-89) and Psal-182 (EM-90). Each isolate was cultivated in FN2 broth medium at 18°C with shaking (100 rpm) until obtaining an OD_600nm_ of 0.2 at the beginning of the exponential phase. Thereafter, nine semi-permeable implants were made using 20 cm length dialysis tubes with a 12 kDa–14 kDa molecular weight cut-off (Spectrum Laboratories Inc., LA, CA, USA) as described by Colquhoun and Sørum (1998). One end was sealed and autoclaved in PBS 1X. Then, each tube was inoculated with a total volume of 5 mL for every bacterial culture (0.25 mL + 0.25 mL for the co-cultures) and sealed with a triple knot. Three main groups of bacterial cultures were used: Psal-013 alone, Psal-182 alone, and Psal-013 (0.25 mL) with Psal-182 (0.25 mL) mixed.

For *in vivo* culturing of *P. salmonis* isolates, nine Atlantic salmon (*S. salar*) post-smolts with an average weight of 1 kg were implanted with one dialysis tube (three fish per group). Fish were kept at the Norwegian Institute for Water Research (NIVA, Solbergstrand, Norway) throughout the experiment. The implants were carefully inserted into the abdominal cavity of the fish through a small 4 cm incision along with a pit-tag for marking and later identification. The cut was then sewn along with three to four sutures through all layers of the tissue. The fish were transferred to a common tank containing seawater at an average temperature of 12.5°C. After 6 days, and before any symptoms could be detected, the fish were euthanized with an overdose of benzocaine (Benzoak 200 mg/mL) for implant removal. The implants were washed with PBS 1×, and the bacterial culture was removed and stored on ice until further use. Norwegian Food Safety Authorities (FOTS ID 26316) approved the animal trial in accordance with the laws and regulations for experiments on live animals in the EU (Directive 2010/637EU) and Norway (FOR-2015-06-18-761).

From every fish, the entire content of the dialysis tube was aseptically collected and centrifuged at 5,000×*g* for 10 min at 4°C for pellet collection. Total RNA was extracted from all samples using the Qiagen RNeasy Mini Kit (Qiagen), according to the manufacturer’s protocol. QIAseq FastSelect−5S/16S/23S (Qiagen) was used according to the manufacturer’s instructions for rRNA depletion. The concentration and quality were measured using a Multiskan Sky Microplate Spectrophotometer (Thermo Fisher Scientific). Library preparation and RNA-seq were performed at the Norwegian Sequencing Center (UiO, Norway) using TruSeq Stranded mRNA library prep and Illumina NovaSeq SP System (150 bp paired-end RNA sequencing).

To process the raw files, the Orion High Performance Computing Cluster at the Norwegian University of Life Sciences (NMBU) was used, according to Morales-Lange et al. (2021). Raw reads from the RNA sequencing were trimmed using BBDuk (v34.56). Then, the clean reads were aligned to *P. salmonis* genome Psal-013 (ASM970875v1—RefSeq assembly accession: GCF_009708755.1) or Psal-182 genome (ASM970941v1—RefSeq assembly accession: GCF_009709415.1) using HISAT (v2.1.0). The fragments mapped were counted with featureCounts (v1.4.6-1) and differentially expressed genes (DEGs) between groups were estimated using SARTools (v1.7.3) package in Rstudio (v4.2.0). Significant DEGs were identified when the adjusted p-value (padj) was <0.05.

### 2.7 Statistical analyses

Data from growth rate, gene expression of target biomarkers and biofilm production were analysed and visualized using GraphPad Prism (v8.0.1). One-way ANOVA and Tukey’s multiple comparison test were used to determine significant differences (p <0.05). The growth rate data were correlated using the Corrplot package (v0.84) (Wei and Simko, 2017) in Rstudio (v4.2.0). Correlations were considered significant when the p-value was <0.05.

## 3 Results

### 3.1 *P. salmonis* co-habitation growth

**Figure 1:**
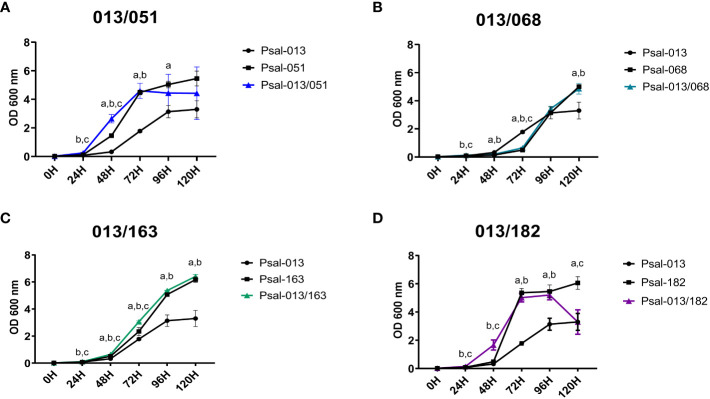
Growth curve of *P. salmonis* isolates in mono- and co-cultures. **(A)** Psal-051 and Psal-013, co-culture in blue. **(B)** Psal-068 and Psal-013, co-culture in light blue. **(C)** Psal-163 and Psal-013, co-culture in green. **(D)** Psal-182 and Psal-013, co-culture in purple. Vertical bars indicate the standard deviation. Letters indicate statistical differences at the same time for different cultures: (a) LF-89-like isolate versus EM-90-like isolate, (b) LF-89-like isolate versus co-culture, (c) EM-90-like isolate versus co-culture.

Growth data showed that in monocultures, the LF-89-like isolate (Psal-013) exhibited the lowest growth of all *P. salmonis* isolates, reaching an OD_600 nm_ of 3.30 after 120 h (**Figures 1A–D**). In comparison, the EM-90 Psal-051 isolate reached an OD_600 nm_ of 5.46 after 120 h (**Figure 1A**). Psal-051 exhibited the shortest lag phase of 24 h among the remaining EM-90 isolates. Interestingly, the co-culture of Psal-013 and Psal-051 showed a decrease in growth after 120 h, reaching an OD_600 nm_ of 4.42.

The monoculture of EM-90 isolate Psal-068 reached the logarithmic phase after 72 h of growth (**Figure 1B**). Psal-068, thereby, had the longest lag phase of all *P. salmonis* isolates, with a peak growth of 5.01 measured at OD_600 nm_ after 120 h. Similar growth was observed when Psal-068 was co-cultured with Psal-013, following the same curve as Psal-068 alone (**Figure 1B**).

Moreover, the monoculture growth of the EM-90 isolate Psal-163 (**Figure 1C**) reached the logarithmic phase after 72 h, and the peak of growth was measured as 6.18 at OD_600 nm_. In the co-culture with Psal-013, no differences were observed in the growth curve (**Figure 1C**).

The EM-90 isolate Psal-182 exhibited the fastest logarithmic growth, reaching the stationary phase after 72 h, and a peak growth of 6.06 measured at OD_600 nm_ after 120 h (**Figure 1D**). In the co-culture experiment with Psal-013, the lag phase was shorter and after 96 h reached a growth of 5.20 at OD_600 nm_, a decline phase started with an OD_600 nm_ of 3.29. For the re-plating results, all cultures were viable, and no contamination was detected at any time (data not shown).

### 3.2 EM-90 and LF-89 growth ratio in co-cultures

**Figure 2:**
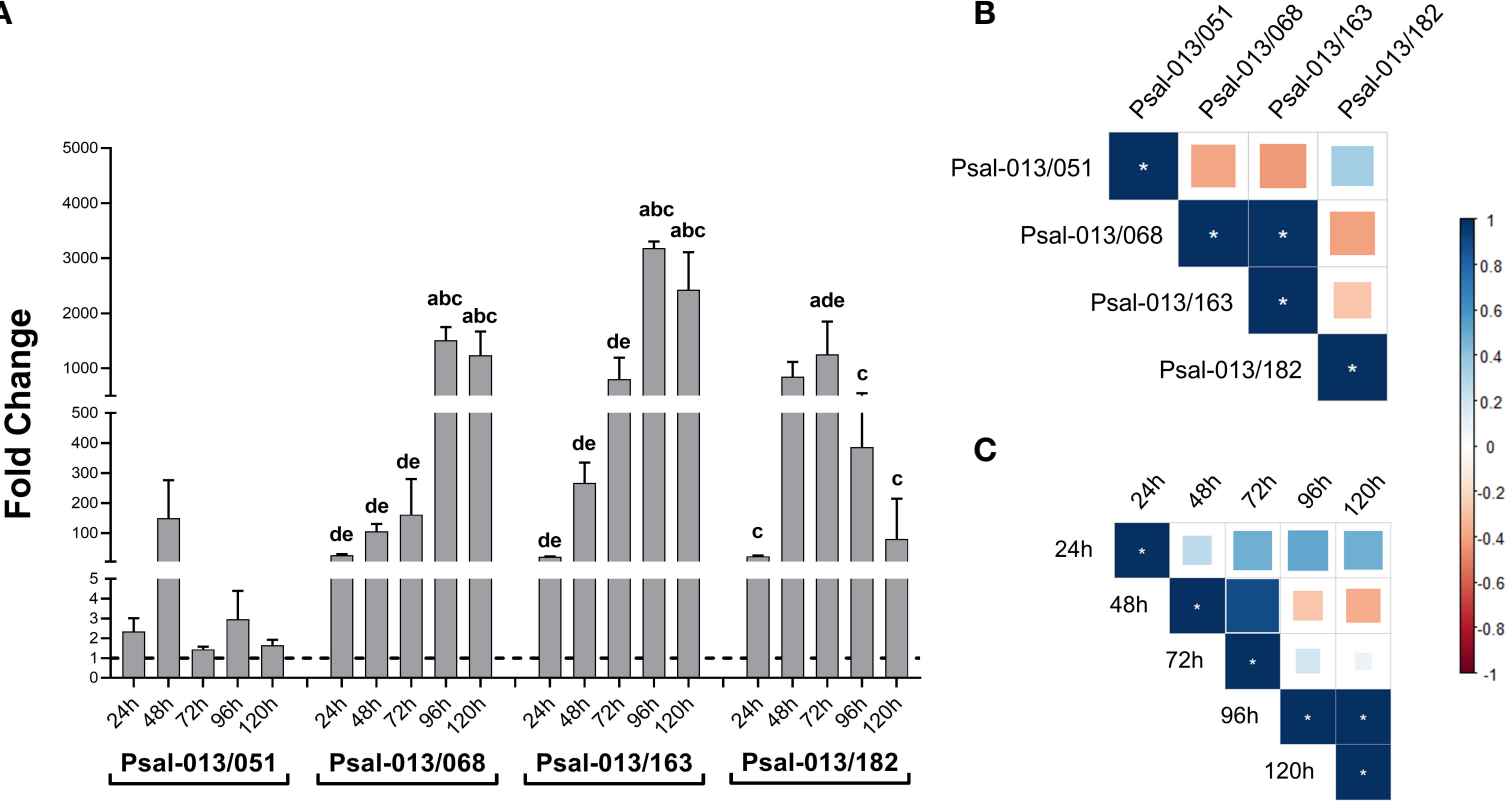
**(A)** EM-90 and L-89 growth ratio during co-cultures with standard deviation. Groups of different lowercase letters above the bar charts of each co-culture indicate significant differences (p <0.05) compared with different times: 24 h (a), 48 h (b), 96 h (c), 120 h (d). **(B)** Correlation coefficient between all isolates. **(C)** Correlation coefficient of co-cultures by time. *: significant values (p <0.05).

After determining the effect of co-culturing the LF-89 isolate (Psal-013) with different isolates from EM-90, the growth ratio of the two genotypes in the co-culture was investigated. The results displayed in **Figure 2A** shows that all EM-90 isolates exhibited improved growth at the expense of LF-89. The Psal-013/051 co-culture showed the lowest difference in fold change. Co-cultures of Psal-013/068 and Psal-013/163 exhibited a similar trend, with a peak in growth after 96 h, with significant differences in time. However, in the co-culture of Psal-013 and Psal-182, the peak was reached at 72 h, after which the ratio between the genogroups began to decline.

When investigating the correlation of all co-cultures, a positive correlation between Psal-013/068 and Psal-013/163 was found (**Figure 2B**). This finding suggests a similar growth ratio for EM-90 and LF-89 in these two co-cultures. Furthermore, the correlation of co-cultures by time showed a positive correlation between 96 h and 120 h (**Figure 2C**), which means that the ratio at 96 h and 120 h remained similar.

### 3.3 Biofilm formation of *P. salmonis* LF-89 and EM-90 genotype and their co-cultivation

As the establishment of biofilms may influence bacterial pathogenesis, it was of interest to explore differences in biofilm formation between the *P. salmonis* LF-89 and EM-90 strains, and when the two genotypes were co-cultivated.

**Figure 3:**
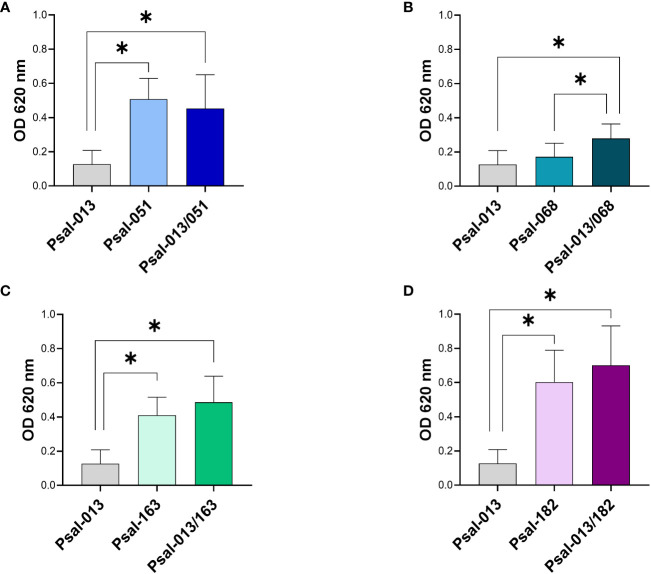
Biofilm production after 120 h of growth. **(A)** Psal-051 and Psal-013. **(B)** Psal-068 and Psal-013. **(C)** Psal-163 and Psal-013. **(D)** Psal-182 and Psal-013. *: significant values (p <0.05).

The results (**Figure 3**) showed that the monoculture of Psal-013 (LF-89-like) had the lowest biofilm production (OD_620 nm_ = 0.123 ± 0.08). While Psal-182 reached a higher biofilm production in co-cultures (OD_620 nm_ = 0.70 ± 0.22) than in monoculture (OD_620 nm_ = 0.60 ± 0.08) (**Figure 3D**). Psal-051 in monocultures, reached a higher production (OD_620 nm_ = 0.507 ± 0.12) than in co-cultures (OD_620 nm_ = 0.45 ± 0.19), but without significant differences (**Figure 3A**). Psal-068 (**Figure 3B**) significantly increased biofilm production from monoculture (OD_620 nm_ = 0.17 ± 0.07) to co-culture (OD_620 nm_ = 0.27 ± 0.08). Although, Psal-163 showed the same trend (**Figure 3C**), a lower biofilm production in monoculture (OD_620 nm_ = 0.41 ± 0.10) than in co-cultures (OD_620 nm_ = 0.48 ± 0.15), there was no significant differences.

### 3.4 *In vitro* co-cultivation influence on gene expression

Co-cultivation of *P. salmonis* LF-89 and EM-90 strains affected bacterial growth phenotypes. Therefore, the next step was to investigate the effects of co-cultivation on gene expression. First we compared the expression of four marker genes (*flaA, relA, luxR* and *cheA* genes) after 120 h of growth. *flaA* encodes the major subunit of flagellin in flagella, while *relA* activates (p)ppGpp, which is involved in regulating growth and different stress responses in bacteria, *luxR* is related to communication through N-acylhomoserine lactone (AHL) quorum-sensing (QS) signaling and *cheA* to chemotaxis.

**Figure 4:**
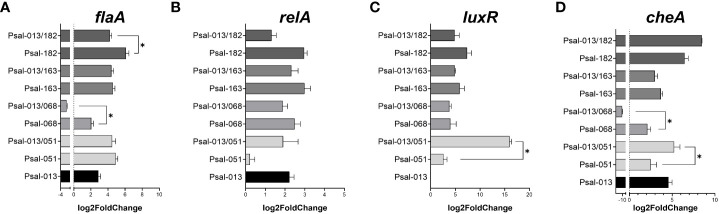
Expression of the marker genes *flaA* **(A)**, *relA* **(B)**, *luxR* **(C)**, and *cheA* **(D)** genes during co-culture after 120 h of growth. *: significant values (p <0.05).

For flagellin (*flaA*) (**Figure 4A**), Psal-182 alone showed the highest expression at 120 h compared with all other cultures, with a significant difference from the co-culture with Psal-013. In contrast, Psal-068 and Psal-013 co-culture showed the lowest expression among all cultures, with a significant difference compared to Psal-068 alone. Psal-051 and Psal-163 in the co-culture presented a similar level of expression to that of Psal-013 grown alone.

*RelA* gene expression (**Figure 4B**) was not significantly downregulated in the Psal-051 monoculture. For *luxR* (**Figure 4C**), a significant difference was observed in the high expression during the co-culture of Psal-051 and Psal-013 compared to Psal-051 monoculture. However, no expression was observed for the LF-89 isolate Psal-013 alone. Moreover, for Psal-068 and Psal-163, there were no significant differences related to their co-cultures.

Psal-013/182 co-culture showed the highest expression of *cheA* but was not significantly higher than that in the Psal-182 monoculture (**Figure 4D**). In addition, Psal-163 in mono- and co-cultures showed no differences in expression related to Psal-013 or between them. In contrast, Psal-051 co-cultured with Psal-013 showed significantly higher expression than Psal-051 monoculture. Finally, the co-culture of Psal-013/068 showed the lowest expression among all the cultures, with a significant difference from the Psal-168 monoculture.

### 3.5 Differential expression of genes during *in vivo* co-cultivation

RNA-seq analysis was performed from the co-cultivation of *P. salmonis* genotypes *in vivo* (within the dialysis bag in the fish). The DEG distribution from each pairwise comparison is shown in **Table 3** (co-culture vs. Psal-182, co-culture vs. Psal-013, and Psal-182 vs. Psal-013). The metadata (normalized gene expression, description, and statistical analysis) are listed in **Table S1**.

**Table 3:** DEGs up and downregulated during *in vivo* culture.

Pairwise comparison	DEGs	
	Upregulated	Downregulated
Co-culture vs Psal-182	12	4
Co-culture vs Psal-013	17	1
Psal-182 vs Psal-013	10	14

Of the 12 upregulated DEGs in the co-culture compared to Psal-182, 11 belonged to the chromosome and one to plasmid 4. Moreover, of the four downregulated DEGs, three corresponded to the chromosome and one to plasmid 4. In the co-culture, compared to Psal-013, 10 upregulated DEGs corresponded to chromosome and seven to plasmid 1. The unique downregulated DEG belongs to chromosome (**Table S1**). Psal-182 monoculture compared to Psal-013 showed seven upregulated DEGs belonging to the chromosome, two to plasmid 4, and one to plasmid 2. In the same pairwise comparison, six of the 14 downregulated DEGs belonged to the chromosome, three to plasmid 4, four to plasmid 2 and one to plasmid 1 (**Table S1**).

Regarding Hypothetical Protein (HP), among the four downregulated DEGs detected in co-cultures compared to Psal-182, two were identified as HP. Similarly, in co-cultures compared with Psal-013, seven upregulated DEGs were identified as HP. In addition, in the Psal-182 monoculture compared to Psal-013, two of the 10 upregulated DEGs were identified as HP and eight out of 14 downregulated DEGs were identified as HP.

**Figure 5:**
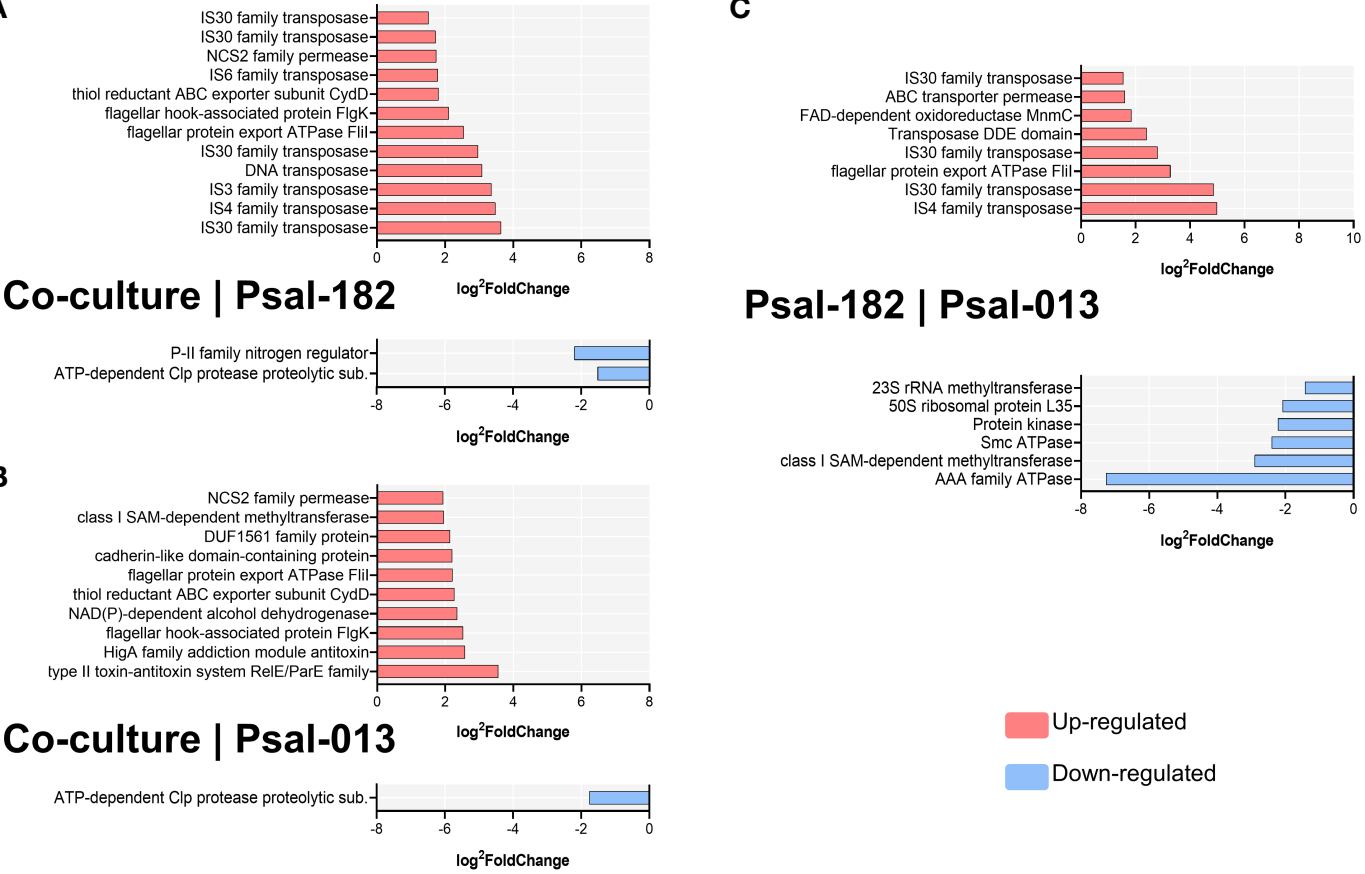
Significant differentially expressed genes (DEGs) per comparison during *in vivo* culturing. **(A)** Co-culture versus Psal-182 comparison. **(B)** Co-culture versus Psal-013 comparison **(C)** Psal-182 versus Psal-013 comparison (in monocultures). Data sorted by log2foldChange. Upregulated in red; Downregulated in blue.

During co-culture, compared to Psal-182, the most upregulated gene was IS30 family transposase (Psal182_RS17815) located in plasmid 4, with 3.641 of fold change, followed by IS4 family transposase (helix-turn-helix domain-containing protein) (Psal182_RS12950) 3.478-fold change, IS3 family transposase (Psal182_RS02820) 3.358-fold change, DNA transposase (Psal182_RS01495) 3.085-fold change, IS30 family transposase (Psal182_RS12275) 2.966-fold change, flagellar protein export ATPase FliI (Psal182_RS02060) 2.547-fold change, and flagellar hook-associated protein FlgK (Psal182_RS10205) 2.114-fold change. The downregulated DEGs were P-II family nitrogen regulator (Psal182_RS15510) with a −2.205 of fold change and ATP-dependent Clp protease proteolytic subunit (Psal182_RS08200) with a −1.521-fold change (**Figure 5A**).

Compared to Psal-013, during co-culture, the most upregulated gene was the type II toxin–antitoxin system RelE/ParE family (Psal013_RS16670) with 3.558-fold change, followed by HigA family addiction module antitoxin (Psal013_RS16660) with 2.581-fold change, flagellar hook-associated protein FlgK (Psal013_RS06465) with 2.522-fold change, NAD(P)-dependent alcohol dehydrogenase (Psal013_RS16675) with 2.354-fold change, thiol reductant ABC exporter subunit CydD (Psal013_RS11795) with 2.268-fold change, flagellar protein export ATPase FliI (Psal013_RS14360) with 2.22-fold change, and cadherin-like domain-containing protein (Psal013_RS10175) with 2.205-fold change. In contrast, the only downregulated DEG was the ATP-dependent Clp protease proteolytic subunit (Psal013_RS08085) with a fold change of −1.767 (**Figure 5B**).

In Psal-182 *in vivo* monocultures compared to Psal-013, the upregulated genes were IS4 family transposase (Psal182_RS12950) with 4.991 of fold change, followed by IS30 family transposase (Psal182_RS17815) (from plasmid 4) 4.86-fold change, flagellar protein export ATPase FliI (Psal182_RS02060) 3.28-fold change, IS30 family transposase (Psal182_RS12275) 2.809-fold change, Transposase DDE domain (Psal182_RS01495) 2.403-fold change, FAD-dependent 5-carboxymethylaminomethyl-2-thiouridine(34) oxidoreductase MnmC (Psal182_RS09240) 1.85-fold change, ABC transporter permease (Psal182_RS11665) 1.609-fold change, and IS30 family transposase (Psal182_RS17360) (located in plasmid 2) 1.552-fold change. The downregulated DEGs were AAA family ATPase (Psal182_RS17775) with a −7.26 fold change, followed by class I SAM-dependent methyltransferase (Psal182_RS01935) with a −2.903-fold change, Smc ATPase (Psal182_RS02460) −2.405-fold change, Protein kinase (Psal182_RS16425) with a−2.225-fold change, and 50S ribosomal protein L35 (Psal182_RS02405) with a −2.087-fold change (**Figure 5C**).

## 4 Discussion

Intensive fish farming has led to the increased occurrence of infectious diseases in aquaculture-related species (Sundberg et al., 2016). Moreover, co-infections in aquatic organisms are natural events that should be considered when studying the biology of pathogens for control measures (Kotob et al., 2016), since infection with multiple pathogens may increase the severity of diseases in fish (Long et al., 2019). Nevertheless, in the case of *P. salmonis* the possibility of a mixed infection of the two genogroups described in Chile has been mostly ignored because the official surveillance program aims to detect the pathogen as a species in salmon farms to prevent and outbreak of SRS, but it does not consider the identification of the genogroups.

In our study, five isolates of *P. salmonis* were chosen based on their genomic features (Mauel et al., 1999), the sub-phylogenetic organization of the genetically diverged EM-90 genogroup composed by four groups, and the LF-89 monophyletic group (Schober, 2022). To evaluate the relevance of this, one isolate belonging to each subgroup was chosen to present a wider view of *P. salmonis* genomes and differences among them under *in vitro* conditions. The results showed evidence of heterogeneity in growth and biofilm production within the EM-90 genogroup. Furthermore, the LF-89 genogroup isolate presented a significantly lower growth rate and biofilm production than all EM-90 isolates. Considering that both genogroups belong to the same species, this supports the differences within the bacterial population of *P. salmonis* that have been described previously (Bohle et al., 2014).

Using FN2 broth medium for bacterial growth, we achieved a shorter lag phase and a longer logarithmic phase compared with previous studies (Yanez et al., 2012). In that report, they achieved an OD_620 nm_ of 1.8 after 6 days using an EM-90-like isolate, while here we achieved a mean OD_600 nm_ of 5.7 measured after 5 days for the four EM-90 isolates in monocultures and a mean OD_600 nm_ of 4.7 for co-cultures due to the lower growth rate of the LF-89 isolate, which had a mean OD_600 nm_ of 3.3 at the same time. This is in line with the evaluated genogroup ratio of growth during co-cultures using specific primers, where the positive correlation between Psal-013/068 and Psal-013/163 indicated a similarity between these two EM-90 isolates. Thus, to obtain higher *P. salmonis* biomass, FN2 broth medium is recommended.

In the growth kinetics we observed in Psal-182 co-cultured with Psal-013, a large amount of biofilm was formed at 120 h, which may have interfered with OD measurements as shown in **Figure 1D**. Biofilm production in *P. salmonis* is a well-described virulence factor (Marshall et al., 2012; Levipan et al., 2020; Santibanez et al., 2020; Zuniga et al., 2020) that is used as a form of survival strategy and resistance to environmental stressors. The results for Psal-013 (LF-89-like isolate), which had the lowest growth rate and biofilm production, suggest that this is the least virulent isolate. Additionally, three EM-90-like isolates (Psal-068, Psal-163, and Psal-182) exhibited higher biofilm production during the co-cultures. Apart from the biofilm level in the co-culture of Psal-051 (EM-90-like) that could be explained by the higher proportion of LF-89 (Psal-013). These data support the evidence that enhanced biofilm formation may be triggered by the synergistic effect of co-culturing *P. salmonis* genotypes, which could be indicative of higher virulence during cohabitation (Levipan et al., 2020) as well as the heterogeneity of different *P. salmonis* isolates (Oliver et al., 2023).

Gene expression of target biomarkers showed that *flaA* (flagellin) was upregulated in monocultures compared to co-cultures with Psal-013, Psal-182, and Psal-068. This is relevant even though this pathogen is described as non-motile, because the flagellin monomer has been detected using a specific antibody under cell-free conditions and during infection in SHK-1 cells (Carril et al., 2017). This suggests that the transcriptional cascade of flagellar genes responds distinctively to co-culture conditions. Regarding *CheA*, a gene related to chemotaxis (Tremaroli et al., 2011) and biofilm production in fish pathogens (Yang and Defoirdt, 2015), our data showed the highest expression in Psal-182, which coincided with the higher production of biofilm. These results are consistent with those of previous studies on *P. salmonis* (Albornoz et al., 2017). Remarkably, *luxR*, which is related to N-acylhomoserine lactone (AHL) quorum-sensing (QS) signaling for bacterial communication (Subramoni and Venturi, 2009; Ruiz et al., 2021), was not expressed in the LF-89 isolate Psal-013. Neverthelss, even though *luxR* is found in the Psal-013 genome, apparently under these *in vitro* conditions is not being expressed.

In *in vivo* cultures, a lower number of DEGs was observed compared to other transcriptome analyses performed in *P. salmonis*. However, they used different approaches to study metabolic resistance to antibiotics (Ortiz-Severin et al., 2021) and virulence during biofilm formation (Levipan et al., 2022). In this study, an *in vivo* culture of *P. salmonis* was used for the first time to study transcriptional changes, and the RNA-seq data showed what is driving the early infection process. Interestingly, in co-cultures, compared to monocultures of the EM-90-like isolate Psal-182, 50% of the most differentially expressed genes corresponded to transposons that function as mobile elements for DNA rearrangements, transcriptional changes, or gene regulation (De Palmenaer et al., 2008; Lysnyansky et al., 2009). In addition, different transposase families were upregulated simultaneously in plasmids and chromosome, such as in IS30 of Psal-182, which could indicate the importance of the plasmids for *P. salmonis* virulence, as described previously (Saavedra et al., 2018).

In addition, the IS6 family transposase was upregulated during co-culture compared to Psal-182, which has been described as relevant to the spread of antibiotic resistance by horizontal transference of genes inside bacterial populations (Varani et al., 2021). A similar pattern was detected for the IS4 transposase family, which triggers important rearrangements in prokaryotic genomes as a tool for genome flexibility and evolution (De Palmenaer et al., 2008), and for DNA-binding transposase (part of Class II transposons), in addition to the IS3 family transposase, which catalyzes the movement of transposons to other parts of the genome by a replicative transposition mechanism (Duval-Valentin et al., 2001). This is interesting because genome rearrangements and deletions linked to transposons have improved the ability of bacteria to combat host defenses, likely by changing surface antigens and regulatory circuitry (Siguier et al., 2014), which is in concordance with previous transcriptomic analysis of *P. salmonis*, where these mobile elements were reported (Nourdin-Galindo et al., 2017; Ortiz-Severin et al., 2019; Valenzuela-Miranda et al., 2020; Levipan et al., 2022). Therefore, the high transcriptional activity of transposases may reflect the stimulus of the environment by using salmon as a living culture broth.

Likewise, the gene encoding the flagellar protein export ATPase FliI was overexpressed during co-culture compared to Psal-182, even though this bacterium is non-motile. However, flagellin monomer synthesis is a well-known virulence effector (Zuniga et al., 2019; Rozas-Serri, 2022). This suggests that FliI can be used for immunomodulation and infection in the host (Carril et al., 2017). Similarly, the flagellar hook-associated protein FlgK, which is required for cell adhesion/invasion in *Cronobacter sakazakii* was upregulated (Li et al., 2023). Another relevant finding is the thiol reductant ABC exporter subunit cydD used for cysteine/glutathione transporter, which is key in the maintenance of redox homeostasis and for correct disulfide bond folding (Shepherd, 2015). This is required for many extra cytoplasmic virulence factors such as the flagellar P-ring motor protein FlgI (Dailey and Berg, 1993), which should act along with FlgK in flagellar assembly. In addition, FlgK is well-characterized in the establishment of pathogenic bacterial infections, to cope with nitric oxide (NO) (Holyoake et al., 2015). The upregulation of NCS2 family permease requires new hypotheses for its function in *P. salmonis*. These molecules have nucleobase transmembrane transporter activity, which has been shown to contribute to virulence in *Francisella tularensis* through attenuated mutants (Meibom and Charbit, 2010), suggesting a role for host colonization during infection.

In co-cultures, compared to Psal-182, the downregulation of the P-II family nitrogen regulator was interesting because this gene is related to nitrogen metabolism control and transcriptional factor activity (Huergo et al., 2013). This suggests a possible mechanism for modulating gene expression during the early stages of infection. In addition, the downregulation of the ATP-dependent Clp protease involved in the proteolysis of defective and misfolded proteins (Aljghami et al., 2022) can be associated with the increased transcription of flagellin-related genes, similar to that described in mutants of *S. typhimurium* with a clpXP deletion (Tomoyasu et al., 2002). Moreover, it has been found to be modulated in *P. salmonis* during SHK-1 cell infection (Figueroa et al., 2021).

When comparing the *in vivo* Psal-182 and Psal-013 monocultures, the ABC transporter permease related to the production of siderophores and iron used by *P. salmonis* (Pulgar et al., 2015; Calquin et al., 2018) was upregulated, supporting its importance for infection in Atlantic salmon. This has been described in *F. tularensis* as being directly related to virulence (Meibom and Charbit, 2010). In addition, in the same comparison, FAD-dependent oxidoreductase MnmC was upregulated. This gene is needed for correct post-transcriptional modifications of tRNA that allows accurate reading of specific mRNA codons during translation on the ribosome (Kitamura et al., 2012; Moukadiri et al., 2018), with a transcription regulatory function. This can also be linked to the upregulation of the same family of transposases during co-culture (IS4 and IS30 from chromosome, plasmid 4, and plasmid 2 of Psal-182), contributing to the relevance of plasmids for virulence.

For the LF-89-like isolate (Psal-013) during co-cultures, there was another set of upregulated genes. For instance, the type II toxin-antitoxin system RelE/ParE family, referred to as an mRNA interferase or inhibitor of protein synthesis, has been linked to the reversible inhibition of cellular metabolism in response to cellular stress (Muthuramalingam et al., 2019). The HigA family antitoxin, that functions as a transcriptional regulator related to virulence factors, such as siderophores, and biofilm production in *Pseudomonas aeruginosa* (Wood and Wood, 2016). Both of these genes are located in plasmid 1 of Psal-013, similar to that described for toxin–antitoxin (TA) systems in other bacteria (Kamruzzaman et al., 2021). This is in concordance with a previous study on *P. salmonis* LF-89 plasmids, which suggests that TA systems contribute to the heritability of the plasmids and carry virulence factors (Ortiz-Severin et al., 2019). Another upregulated DEG in the same plasmid was the DUF1561 family protein, which was described as part of Virulence-Modifying (VM) proteins in *Leptospira*, and may be involved in the secretion of exotoxins with virulence effects (Vieira et al., 2023).

The upregulation of cadherin-like domain-containing proteins is relevant, since it is pivotal for adherence to host cells, mediating a well-studied host–pathogen interaction (Dash et al., 2021) for *P. salmonis* during biofilm formation (Levipan et al., 2022). This could be a key virulence factor for invasion, which was only upregulated by Psal-013 when cohabiting with Psal-182. Additionally, the genes encoding the flagellar hook-associated protein FlgK, the thiol reductant ABC exporter subunit CydD, and the NCS2 family permease were also upregulated for Psal-013 during the *in vivo* co-culture, along with the downregulation of the Clp protease. Their increased expression *in vitro* indicates the importance of these virulence effectors in adhesion or early infection processes and is triggered by the cohabitation of both genogroups. Their relevance should be addressed in further studies on the pathogenesis of *P. salmonis*.

In summary, the gene expression pattern was modulated differently by each genogroup during *in vivo* co-culture. Psal-013 (LF-89) displayed the expression of more DEGs described as virulence factors than Psal-182 (EM-90) under the co-culture condition, which suggest that the cohabitation might play a role in the pathogenesis of SRS. Therefore, it is relevant to consider these within-host competitive interactions because they are linked to virulence and can also modulate the dynamics of the infection process. The different antigenic epitopes present in this mixed population can trigger a cross-reactive immune response in the host (Balmer and Tanner, 2011; Kinnula et al., 2017), altering the immune efficiency and welfare of fish.

## 5 Conclusions

In this study, we have shown that the co-habitation of the LF-89 and EM-90 genogroups of *P. salmonis* induced changes in growth and biofilm production during *in vitro* co-cultures, indicating a synergistic effect. The differential expression of virulence factors observed under *in vivo* co-culture may be related to increased pathogenicity to the host. These effects will be studied in further research with co-infection challenges in Atlantic salmon to evaluate the disease dynamics under field conditions. Likewise, we propose new candidate genes used by *P. salmonis* as virulence effectors (*cydD*, *flgK*, and *NSC2*). Furthermore, we demonstrated that the use of specific primers for RT-qPCR is an efficient strategy for identifying the *P. salmonis* genogroups. Finally, this approach to SRS as a multiple-genotype infection may offer better insights for proposing novel disease control measures.

## Data availability statement

The datasets presented in this study can be found online in Gene Expression Omnibus (NCBI): GSE235725. **Supplementary Material** can be found in the article.

## Ethics statement

The animal study was approved by the Norwegian Food Safety Authorities (Mattilsynet), Norway, FOTS ID 26316. The study was conducted in accordance with the local legislation and institutional requirements.

## Author contributions

GC: Conceptualization, Data curation, Formal Analysis, Funding acquisition, Investigation, Methodology, Visualization, Writing – original draft, Writing – review & editing. HW-L: Conceptualization, Methodology, Supervision, Writing – review & editing. ML: Conceptualization, Supervision, Writing – review & editing. HS: Conceptualization, Funding acquisition, Methodology, Project administration, Resources, Supervision, Writing – review & editing.
